# Identification of further variation at the lipooligosaccharide outer core locus in *Acinetobacter baumannii* genomes and extension of the OCL reference sequence database for *Kaptive*

**DOI:** 10.1099/mgen.0.001042

**Published:** 2023-06-13

**Authors:** Bianca M. Sorbello, Sarah M. Cahill, Johanna J. Kenyon

**Affiliations:** ^1^​ Centre for Immunology and Infection Control, School of Biomedical Sciences, Faculty of Health, Queensland University of Technology, Brisbane, Australia

**Keywords:** *Acinetobacter baumannii*, Kaptive, lipooligosaccharide, OCL, OC locus

## Abstract

The outer core locus (OCL) that includes genes for the synthesis of the variable outer core region of the lipooligosaccharide (LOS) is one of the key epidemiological markers used for tracing the spread of *Acinetobacter baumannii *, a bacterial pathogen of global concern. In this study, we screened 12 476 publicly available *A. baumannii* genome assemblies for novel OCL sequences, detecting six new OCL types that were designated OCL17–OCL22. These were compiled with previously characterized OCL sequences to create an updated version of the *A. baumannii* OCL reference database, providing a total of 22 OCL reference sequences for use with the bioinformatics tool *Kaptive*. Use of this database against the 12 476 downloaded assemblies found OCL1 to be the most common locus, present in 73.6 % of sequenced genomes assigned by *Kaptive* with a match confidence score of good or above. OCL1 was most common amongst isolates belonging to sequence types (STs) ST1, ST2, ST3 and ST78, all of which are over-represented clonal lineages. The highest level of diversity in OCL types was found in ST2, with eight different OCLs identified. The updated OCL reference database is available for download from GitHub (https://github.com/klebgenomics/Kaptive; under version *v. 2.0.5*), and has been integrated for use on *Kaptive-*Web (https://kaptive-web.erc.monash.edu/) and PathogenWatch (https://pathogen.watch/), enhancing current methods for *A. baumannii* strain identification, classification and surveillance.

## Data Summary

1. The updated *Acinetobacter baumannii* OCL reference sequence database including 22 annotated OCL sequences is available for download under *Kaptive v. 2.0.5* at https://github.com/klebgenomics/Kaptive.
2. Genome assemblies or GenBank records used as representative reference sequences are listed in Table 1 and acknowledged in each record in the database.

**Impact Statement**

In the absence of effective treatment options for multi-drug resistant *Acinetobacter baumannii,* the highest-ranking critical priority bacterial pathogen of global concern, national and global surveillance is necessary to detect, track and subsequently curb the spread of isolates that resist current therapies. Several epidemiological markers are used to characterize *A. baumannii* strains by detecting genetic differences in specific regions of the genome. One of these is the chromosomal outer core locus (OCL) responsible for the synthesis of the outer core (OC) component of the lipooligosaccharide (LOS). Here, we provide an update to the international *A. baumannii* OCL reference sequence database, extending the number of known OCL types to assist with clinical surveillance of important strains or clonal lineages.

## Introduction

*Acinetobacter baumannii* is a Gram-negative coccobacillus that is recognized as one of six leading pathogens responsible for nearly three-quarters of deaths associated with antibiotic-resistance worldwide [1]. The species has been detected in most geographical regions around the world and has been found to account for more than one-fifth of all hospital-acquired infections in Europe, the Eastern Mediterranean and Africa [2]. The World Health Organization has ranked carbapenem-resistant *A. baumannii* as a ‘Priority 1: CRITICAL’ bacterial pathogen [3], and with an estimated 80 % of circulating isolates now resistant to last-line carbapenems [1], new therapeutic approaches are urgently needed. However, in the current absence of widely accessible, approved and effective treatments*,* local and international surveillance initiatives are required to control the continued spread of pan-resistant *A. baumannii* isolates.

Whole-genome sequencing (WGS) represents a readily accessible standard for tracking the spread and evolution of resistance in *A. baumannii *. As the spread of carbapenem resistance has been associated with the global dissemination of two major clonal complexes [4], known as global clone 1 (GC1) and global clone 2 (GC2), multilocus sequence typing (MLST) is commonly used in the primary stages of strain characterization to identify the clonal lineage. Two MLST schemes, Institut Pasteur (IP) and Oxford (OX), are available for *A. baumannii *, and GC1 and GC2 include predominately sequence type (ST) 1 and ST2 in the IP scheme [5]. However, as clones continue to evolve and separate into distinct sublineages with different resistance profiles [6–10], two additional epidemiological markers can be used in combination with MLST to further discriminate isolates. These are the K locus (KL) for synthesis of the capsular polysaccharide (CPS), and the outer core locus (OCL) for synthesis of the outer core (OC) of the lipooligosaccharide (LOS) [11–14]. Both CPS and LOS are important cell-surface structures and virulence determinants for *A. baumannii *, and differences in the genetic content at the chromosomal K and OC loci can lead to structural changes in these complex surface molecules [11, 15, 16].

To simplify the ability to detect genetic differences at these loci, a user-friendly nomenclature system that assigns a KL or OCL number to any new combination of genes found at these loci was developed [11]. In 2020, the many different clusters of genes identified at these locations were compiled into KL and OCL reference databases compatible with the bioinformatics search tool *Kaptive* [12]. This tool assigns a best match locus type to queried genome assemblies by screening individual genomes against these databases [17]. The first release of these databases included 92 KL and 12 OCL types, which are available in *Kaptive* versions *0.0.7–2.0.0* [12]. We recently performed a major update to the *A. baumannii* KL reference sequence database releasing a further 149 KLs in version *2.0.1* [13]. However, while a further four OCL types (OCL13–OCL16) have been characterized and reported since the release of the original databases [18, 19], the OCL reference sequence database has not been updated.

**Fig. 1.**
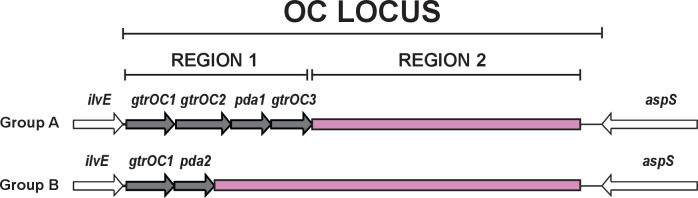
Typical arrangement of gene clusters belonging to group A and group B located at the *A. baumannii* chromosomal OCL for LOS OC biosynthesis. Conserved *ilvE* and *aspS* genes that flank the OCL are shown in white. Grey genes are those common to OCLs belonging to either group A or group B, whereas the pink segments are regions that commonly vary in gene content between different OCL types.

Unlike many Gram-negative bacterial pathogens, *A. baumannii* does not produce a structurally variable O-antigen polysaccharide attached to the OC of the LOS by a WaaL ligase [11, 14, 15] to form an extended structure known as lipopolysaccharide (LPS). Structural variation is, therefore, predominately observed in the OC component, which is directed by differences in the genetic content at the OCL [14, 15]. The OCL is located between conserved *ilvE* and *aspS* genes in the *A. baumannii* chromosome [11], and interruption of genes at this location has been shown to result in truncations to the core component of the LOS structure [15]. All OCLs characterized to date include predominately genes that encode glycosyltransferase enzymes (*gtr*) for linking sugars together to grow the oligosaccharide OC structure, though some OCLs include additional genes for sugar biosynthesis and/or modification [14]. Previously, it was found that the first 12 OCL types identified could be separated into two major genetic arrangements, referred to as group A and group B, respectively, based on the presence of either a *pda1* or *pda2* gene near the start of the locus (Fig. 1). These genes are in ‘region 1’, which also includes a few further genes (*gtrOC1*, *gtrOC2* and *gtrOC3* in group A, and *gtrOC1* in group B) that are shared by all OCL sequences that fall into each group. Diversity in gene content that distinguishes OCL types has so far always been observed in ‘region 2’ (Fig. 1).

Here, we provide an update to the *A. baumannii* OCL reference sequence database to include the four additional OCLs [18, 19] not included in the original version, and any new OCL types that were identified in a search of more than 12 000 publicly available *A. baumannii* genome sequences. Additionally, we define the general characteristics of OCL sequences and assess the distribution of OCL types amongst sequenced isolates, revealing the extent of OCL diversity in important clonal lineages.

## Methods

### *A. baumannii* genome assemblies

Publicly available genome assemblies (*n*=12 553) listed under the *A. baumannii* taxonomical classification (ID: 470) in the National Center for Biotechnology Information (NCBI) non-redundant and WGS databases were downloaded for local analysis (21st of April 2022). To confirm the taxonomical assignment, blastn was used to screen all downloaded genome assemblies for the presence of the *A. baumannii-*specific *oxaAb* gene (also known as *bla*_OXA-51-like_), as described previously [12, 13]. Only genomes confirmed to carry this gene*,* as defined by >90 % combined coverage and >95 % nucleotide sequence identity to the query *oxaAb* gene from *A. baumannii* isolate A1 (GenBank accession number CP010781.1, base positions 1 753 305 to 1 754 129) were used for further analyses.

### Detection and annotation of novel OCL sequences in *A. baumannii* genomes

Using the command-line version of *Kaptive v. 2.0* [20], confirmed *A. baumannii* genomes (*n*=12 476) were screened against an in-house version of the OCL database, which included OCL1–OCL12 reference sequences available in the published database (released under *Kaptive* versions *0.0.7–2.0.4*; https://github.com/klebgenomics/Kaptive) plus the additional four OCL sequences (OCL13–OCL16) [18, 19] identified since its release. Default parameters were used for all *Kaptive* searches, except that the minimum identity cut-off for gene searches using tblastn was specified as 85 % in line with the accepted cut-off for identification and annotation of OCL genes in *A. baumannii* [11, 12].

Using the database, *Kaptive* assigns a ‘best match locus’ to each genome assembly and provides a categorical measure of match confidence, as described elsewhere [12, 13, 17]. These categories are: ‘perfect’ (locus is found in a single piece with 100 % coverage and 100 % identity to a reference sequence); ‘very high’ (locus is in a single piece with ≥99 % coverage and ≥95 % identity to a reference; no missing genes and no extra genes); ‘high’ (locus is in a single piece with ≥99 % coverage to a reference; ≤3 missing genes and no extra genes); ‘good’ (locus has ≥95 % coverage to a reference; with ≤3 missing genes and ≤1 extra genes); ‘low’ (locus has ≥90 % coverage; ≤3 missing genes and ≤2 extra genes); and ‘none’ (did not meet the criteria for inclusion in the other listed categories). All genomes assigned a match with a confidence level less than perfect were further examined. Matches with <95 % total coverage and/or <95 % total nucleotide sequence identity to the best matched sequence, additional or missing genes, and/or length discrepancies were targeted for detailed manual inspection, as described previously [12, 13].

OCL sequences predicted to be novel were visually compared to the assigned best match locus using the pairwise sequence comparison tool, Easyfig *v. 2.2.5* [21]. Default parameters were used for the alignment, except for the minimum tblastx identity cut-off, which was set to 85 %. If additional genes were detected by comparison to the best match locus, ISFinder (https://isfinder.biotoul.fr/about.php) was used to initially determine whether the sequence was an insertion sequence (IS). OCLs that differed only from the best match locus due to the presence of an IS were considered a ‘variant’ of the reference locus and were not further examined, as described previously [12, 13].

In cases where OCLs were found to differ from the best match locus in the presence or absence of other genes, the sequence was considered novel and assigned a new OCL number according to the Kenyon and Hall nomenclature system for OCL typing [11, 12]. The boundaries of each locus were confirmed via the identification of the *ilvE* and *aspS* genes flanking the sequence in the reference genomes using blastn with default parameters. For novel genes found to share <85 % aa sequence identity with known *A. baumannii* OCL gene products, blastp was used to predict their role in OC synthesis by searching for gene homologues of known or predicted function and *hmmscan v. 2.41.2* from the hmmer package [22] was used with the Pfam database to detect any known protein motifs/domains. Novel gene products were assigned new names indicating enzyme function (e.g. GtrOC# for predicted glycosyltransferases) in accordance with the nomenclature system [11, 12].

### Creation and validation of an updated *A. baumannii* OCL reference sequence database

A reference GenBank format (.gbk) file was generated for each novel OCL sequence (details for each record are shown in Table 1), which included the complete nucleotide sequence of the locus and annotations of all coding sequences within that were assigned using the nomenclature scheme [11]. As only one OC structure, OC1, is known for *A. baumannii* [23], an additional note field was added to the OCL1 record to define the OC type as is required for the newest iteration of the *Kaptive* code [20]. For all other OCLs where no OC structural data is available, the note field specifies the structural type as unknown.

**Table 1.** Information on *A. baumannii* OCL reference sequences used to populate the updated OCL database for *Kaptive v. 2.0.5*

OC locus	Reference isolate	GenBank or WGS accession no.	Base range	Length (bp)	No. of ORFs
OCL1	A1	CP010781	3 366 405–3 375 181	8658	9
OCL2	D36	CP012952	646 907–655 270	8307	9
OCL3	A85	CP021782	3 464 825–3 473 737	8449	9
OCL4	A388	CP024418	642 040–650 673	8634	9
OCL5	G21	MG231275	37 120–46 166	9048	9
OCL6	D46	KF030679	28 675–37 977	9303	10
OCL7	OIFC035	AMTB01000038	221 522–230 586	9065	9
OCL8	OIFC111	AMFY01000013	222 496–228 777	6282	6
OCL9	Naval-72	AMFI01000027	34 336–40 843	6508	7
OCL10	AB_TG27343	AMIS01000032	97 841–111 169	10 504	11
OCL11	TG22204	ASFV01000009	33 588–43 841	7104	8
OCL12	NIPH 410	ATGJ01000006	47 175–57 655	10 481	11
OCL13	MRSN7113	OK052579	1–7594	7594	7
OCL14	MRSN14237	OK052580	1–10 808	10 808	11
OCL15	MSHR_A82	DADBCZ010000019	17 081–26 746	9666	10
OCL16	MSHR_A192	DADBCG000000000	101 631–108 716	7086	8
OCL17	Ab-C63	CP051866	561 025–571 697	10 673	10
OCL18	BA7738	JAAOQP010000015	71 442–80 878	6612	6
OCL19	4300STDY7045779	UFKV01000001	1 562 697–1 574 290	8426	9
OCL20	PR310	NGCA01000023	67 660–78 973	8139	9
OCL21	ABBL063	LLFI01000256	73 213–81 386	8174	8
OCL22	ARLG1902	NGIB01000010	293 169–303 755	7392	8

Reference .gbk files for all OCLs were then concatenated into the same file to create an updated OCL database compatible with command-line *Kaptive*, which was released at https://github.com/klebgenomics/Kaptive under version *v. 2.0.5*. The updated database was also integrated with the *Kaptive-Web* (https://kaptive-web.erc.monash.edu/) and PathogenWatch (https://pathogen.watch/) platforms. Over the course of this analysis, a problem was identified that led to the confusion of assignments for the closely related OCL5 and OCL13, and OCL1 and OCL18 loci. The problem was rectified through an update to the *Kaptive* code, which was released with the updated OCL database in *version 2.0.5*. To validate database functionality, all genome assemblies were searched again using *Kaptive v. 2.0.5* with the same parameters as described above.

### Characterization of the genetic repertoire of *A. baumannii* OCL sequences

Prokka *v. 1.13* [24] was used to generate .gff3 files for each OCL reference sequence by directing the designation of gene names to annotations available in each .gbk record in the database. The .gff3 files were used to manually tabulate the sequence lengths and number of ORFs for each OCL type (see Table 1). A gene presence/absence matrix based on a minimum percentage identity of 85 % for blastp was also created using the pan-genome analysis software Roary *v. 3.13.0* [25]. Tabulated data was then visualized using the ggplot2 package in RStudio *v. 3.3.0+* [26]. A representative sequence from each gene homology group was submitted to *hmmscan v. 2.41.2* [22] to re-assess protein family designations for the reported annotations. To further confirm that a gene for a potential WaaL ligase was not located between *aspS* and *tonB* in *A. baumannii *, all genomes were also screened for an insertion of additional sequence present at this location.

### Clonal analysis

MLST was conducted on genome assemblies to assign ST using the MLST tool available at https://github.com/tseemann/mlst with the *A. baumannii* IP scheme. ST groups that included ≥100 genome representatives were identified as over-represented clonal groups, and the percentage of OCL types with a match confidence of good or above within each group was calculated and visualized as a heatmap using RStudio *v. 3.3.0+* with the ggplot2 *v. 3.3.6* package [26].

## Results

### Screening for *A. baumannii* genome assemblies that carry novel OCLs

Confirmed *A. baumannii g*enome assemblies (*n*=12 476) were screened against an in-house version of the OCL reference database that includes the 12 OCLs available in *Kaptive v. 0.7.0–2.0.0* plus the additional OCL13–OCL16 sequences [18, 19] identified since its release. As *Kaptive* assigns a confidence score to each query genome using a categorical measure of match quality (confidence scores described in Methods and in published work [12, 13, 17, 27]), this was used as a marker to identify genomes that may carry novel OCL sequences. The match confidence scores for genomes screened with the in-house database were: 308 (perfect), 8856 (very high), 249 (high), 2567 (good), 94 (low) and 402 (none) (raw data in Table S1, available with the online version of this article). The 308 genome assemblies that were assigned a match confidence level of perfect, indicating that the detected locus is identical to a known OCL reference sequence, were disregarded. Of the remaining genome assemblies, a total of 9166 genomes [73.5 % of the total genome pool; assigned as very high (*n*=8835), high (*n*=166) or good (*n*=165)] were found to be assigned to the correct locus (≥99 % coverage; ≥88 % DNA sequence identity), differing from a reference sequence in SNPs across the length of the locus and/or small insertions/deletions (between −187 and +14 bp) in coding or non-coding regions.

An additional 2834 genomes [22.7 % of the total genome pool, assigned either good (*n*=2346), low (*n*=94) or none (*n*=394) matches] were also not further examined as the detected OCL was identified across two or more contigs, suggesting either low sequence/assembly quality or IS interruptions in the OCL, as described previously [12, 13]. Manual inspection of the remaining genome assemblies (*n*=168) revealed that 24 genomes (6 high, 12 good, 6 none) included multiple gaps (>60 bp), insertions/deletions involving coding sequences, strings of ‘N’ bases, or had been later excluded from NCBI RefSeq due to ‘many frameshifted proteins’ or ‘fragmented assembly’. Hence, these were considered poor-quality sequence or assemblies and removed from further analysis. In addition, two other genomes (NCBI assembly accession numbers GCA_001862305.1 and GCA_001863295.1) were assigned a match confidence score of none with zero of nine expected genes identified. On close inspection of these sequences, an OCL was not present.

A total of 104 genomes (21 very high, 51 high and 32 good) were considered variants of known loci as these were found to carry one or more IS interrupting the locus, indicated by significant discrepancies in the total length of the match (>800 bp of additional sequence). For nine genomes assigned a high confidence match, ISs were found as part of one of two different novel transposons inserted at different locations in the OCL1 locus (shown in Fig. S1). One of these transposons was identified in two different locations, interrupting either *orf1 (ghy*) in seven genomes or *gtr6* in a single genome. It was found to carry a gene encoding an osmotically inducible protein C (OsmC) and a gene predicting a Vrf cAMP receptor protein, flanked by IS*Aba1* transcribed in opposite directions. The other transposon interrupted the *gtrOC7* gene in a single genome and carried genes predicting a lipid A export ATP-binding/permease, a haemolysin D family efflux transporter periplasmic adaptor subunit, a cupin-like domain-containing protein, an Arc family DNA-binding protein and an IS*21* sequence. The predicted gene products from both transposons appear unlikely to influence OC biosynthesis, and while additional sequence was detected between *ilvE* and *aspS*, these variants were not assigned new OCL names.

For 26 genomes that were assigned a match confidence of high, all had been assigned to OCL5 (100 % coverage; 90–91 % DNA sequence identity) but *Kaptive* had detected the presence of a *gtrOC7* glycosyltransferase gene. On close inspection of these loci, *gtrOC7* was found at the terminal end of the locus downstream of *aspS*. As this was a detectable difference in gene content between *ilvE* and *aspS* from OCL5, it was considered a new type and the locus was assigned the name OCL17. The remaining 12 genomes were found to have <91 % DNA sequence coverage, significant length discrepancies (>500 bp), missing expected genes and/or presence of unexpected genes in the locus sequence. Amongst these genomes, five novel OCL types were identified, and these were designated OCL18 (two genomes; good matches), OCL19 (one genome; good match), OCL20 (one genome; good match), OCL21 (five genomes; good matches) and OCL22 (three genomes; good matches). Only four new genes were identified amongst the novel OCL sequences, present in either OCL20, OCL21 or OCL22. Three were predicted to encode glycosyltransferases (named *gtrOC34–36*) and one (named *ORF4*) was predicted to encode a protein of unknown function (see below).

### Organization of new OCLs

**Fig. 2.**
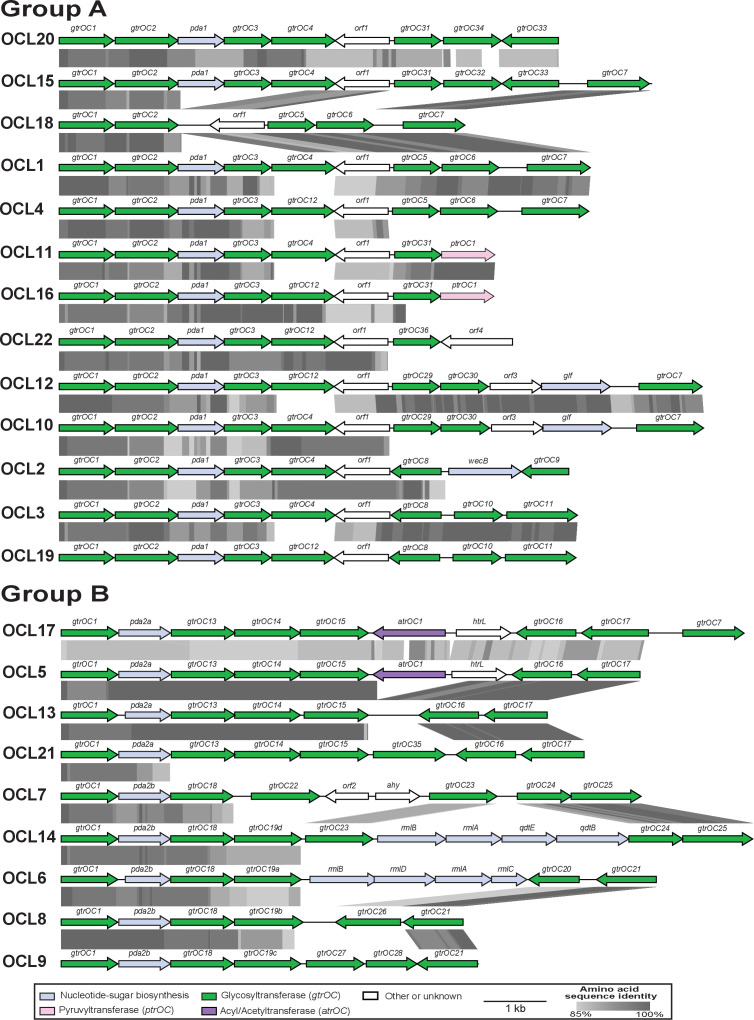
Alignment of all identified OCL reference sequences (OCL1–OCL22) separated into group A and group B. Genes are represented by arrows orientated in the direction of transcription that are coloured by the predicted function of their gene products (shown in the key below the figure). Grey shading between gene clusters shows amino acid sequence identities determined by tblastx with the grey scale shown in the key below. Figures drawn to scale using Easyfig [21] and annotated/coloured in Adobe Illustrator.

All novel OCLs consisted of similar genetic arrangements to their best match locus assigned by *Kaptive*, differing only in the replacement, insertion, or deletion of one, two or three genes (Fig. 2). OCL19, OCL20 and OCL22 included *pda1* and were classified as group A, whereas OCL17 and OCL21 included *pda2* and were classified as group B (Fig. 2). The OCL18 did not include either *pda1* or *pda2,* indicating that it may represent a new group. However, upon alignment of the new OCLs (OCL17–OCL22) with those characterized in previous studies (Fig. 2), OCL18 was found to be a deletion variant of OCL1 missing the *pda1*, *gtrOC3* and *gtrOC4* genes, and was designated as group A. Hence, group A consists of a total of 13 OCLs that all include *gtrOC1*, *gtrOC2* and *orf1,* whereas group B consists of 9 OCLs that all include *gtrOC1* and *pda2*.

Across the set of 22 OCLs, OCL13 (group B) and OCL18 (group A) appear to have arisen via the deletion of specific sequence from the central portion of OCL5 and OCL1, respectively. The close relationship between OCL5 and OCL13 had been described in a previous study [28], for which OCL13 was shown to lack the *atrOC1* gene and a complete copy of the adjacent *htrL* gene that are present in OCL5 (Fig. 2). Alignment of OCL5 and OCL13 revealed the presence of a 4 bp repeat (GTAA) in OCL5 on either side of the deleted region in OCL13, and a single copy of this 4 bp string in OCL13 at the precise location of the deletion (see Fig. S2a). Similarly, a pairwise sequence alignment of OCL1 and OCL18 identified a 4 bp repeat (CTAG) in OCL1 on either side of the deleted region, with a single copy found in OCL18 (Fig. S2b). This finding may suggest how one type arose from another, though further work is needed to confirm the effect on the LOS structure.

### Validation of the updated OCL reference sequence database

**Fig. 3.**
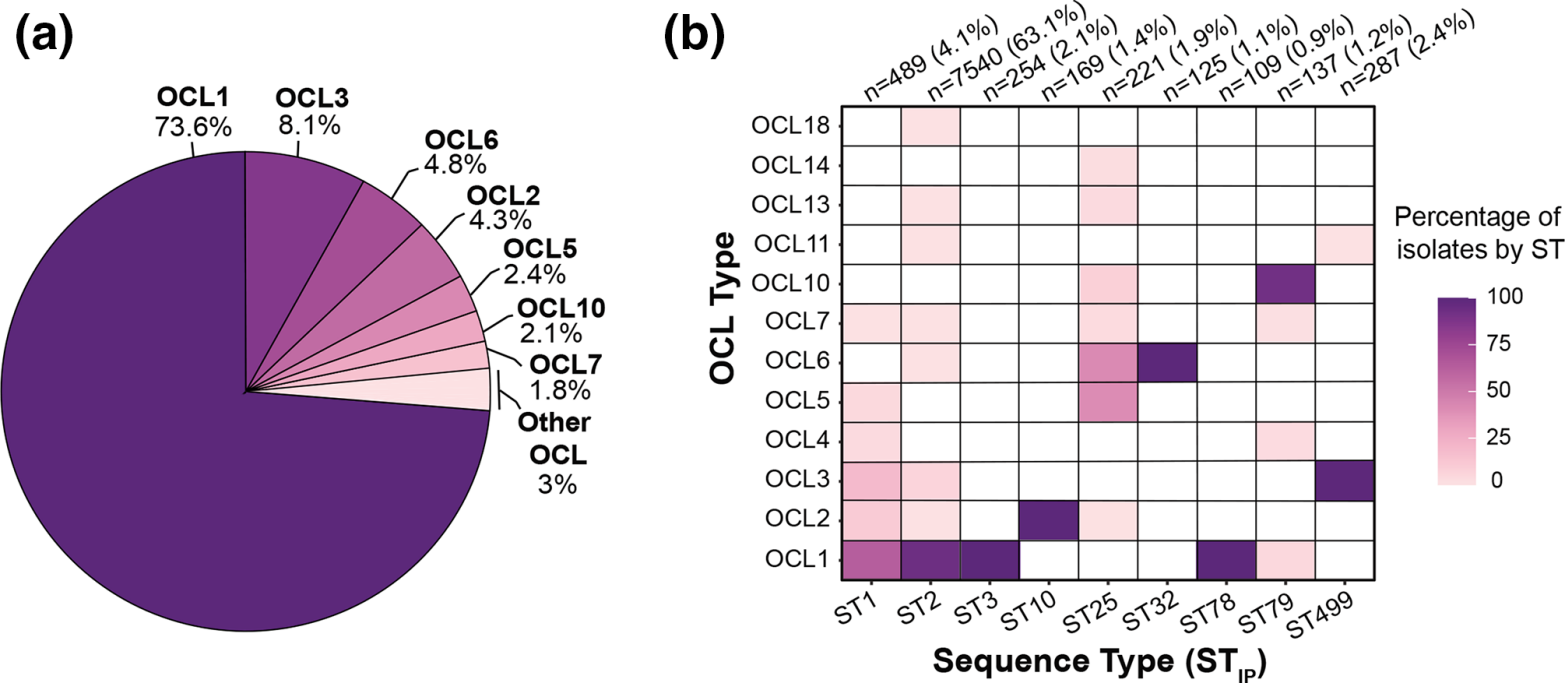
Distribution of OCL types amongst *A. baumannii* genome assemblies. (**a**) Percentage of OCL best match locus types assigned a confidence score of good or above by *Kaptive* (*n*=11 940). (**b**) Heat map showing percentage of genomes belonging to nine over-represented STs that carry OCL best match locus types assigned a confidence score of good or above by *Kaptive*. Only STs with ≥100 genomes are shown (total number of genomes=9331: 489 ST1, 7540 ST2, 254 ST3, 169 ST10, 221 ST25, 125 ST32, 109 ST78, 137 ST79, 287 ST499). Figures were created using the ggplot2 package in RStudio [26].

The six newly identified OCL types were added into an updated reference database, which included concatenated .gbk reference files for all known OCL types (OCL1–OCL22). To validate its use, the updated database was applied to the complete pool of 12 476 genomes, and the match confidence scores obtained were: 344 (perfect), 9031 (very high), 238 (high), 2347 (good), 114 (low) and 402 (none) (Table S2). Using the output of this search (Table S2), the frequency of each OCL was calculated amongst the genomes with an assigned match confidence score of good or above (*n*=11 940). OCL1 was found to be the most common type (Fig. 3a), accounting for 73.6 % (*n*=8782) of the genome pool. A further 23.4 % of genomes (*n*=2797) included one of six other OCL types [OCL3 (8.1 %), OCL6 (4.8 %), OCL2 (4.3 %), OCL5 (2.4 %), OCL10 (2.1 %) and OCL7 (1.8 %)], and together with OCL1 represent 97.2 % of the genomes analysed. The remaining 3 % of genomes (*n*=361) contained one of the other 15 OCL types, each detected at a frequency of <1 %.

### OCLs in clonal genomes

The distribution of OCLs amongst genomes from clonal isolates was also investigated amongst the 11 940 genomes with match confidence scores of good or above. STs represented by 100 or more genome assemblies were identified, revealing nine clonal lineages (ST1, *n*=489; ST2, *n*=7540; ST3, *n*=254; ST10, *n*=169; ST25, *n*=221; ST32, *n*=125; ST78, *n*=109; ST79, *n*=137; and ST499, *n*=287) that were over-represented in this genome pool. ST2 represented 63.15 % of these genomes, followed by ST1 representing 4.1 % of genomes (Fig. 3b). The calculated percentage of clonal isolates that carry each of the OCL types (Fig. 3b) revealed that the predominant ST1 and ST2 lineages include six and eight different OCL types, respectively, with OCL1 being the most common in both clones. The emerging, globally distributed clonal lineage ST25 [4, 29] demonstrated a similar level of diversity at the OCL, with seven OCL types detected, for which OCL5 and OCL6 were the most common types. Only one OCL was detected in ST10, ST3, ST32 and ST78 lineages. Of the novel OCL sequences*,* OCL18 was identified at a low frequency in ST2, while OCL13 and OCL14 were detected in ST25 only.

### General characteristics of sequences at the OCL

**Fig. 4.**
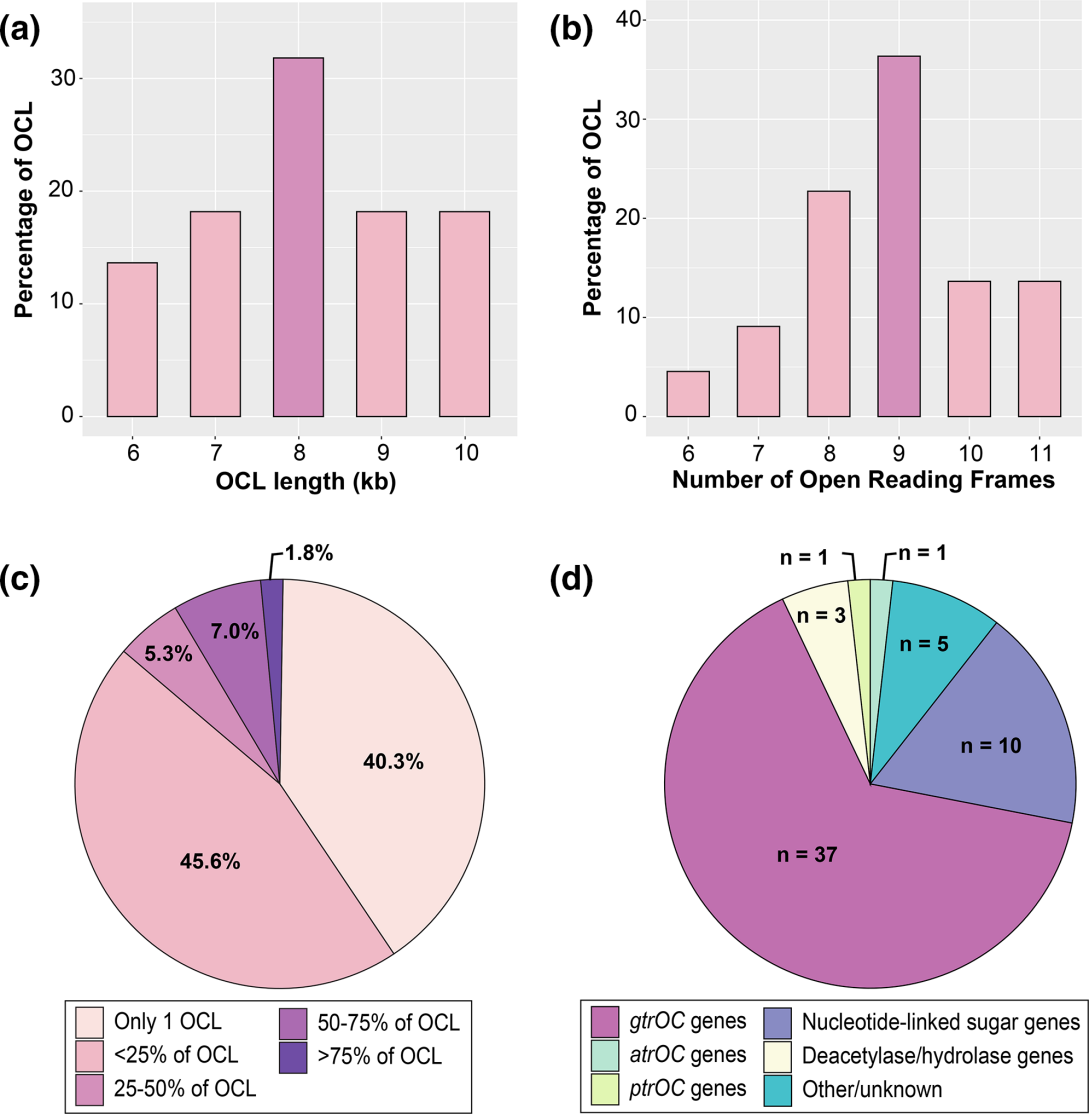
General features of the 22 OCL sequences included in the database. (**a**) Percentage of OCLs per total sequence length (kb). (**b**) Percentage of OCLs that include specified number of ORFs. (**c**) Frequency of 57 gene homology groups found across 22 OCLs as determined by Roary gene presence/absence analysis. The colour scheme is shown in the key below the figure. (**d**) Breakdown of gene types at the OCL found across 22 OCLs. Figures were created using the ggplot2 package in RStudio [26].

To better understand the diversity at the OCL, the general features of all OCL types, including the mean length, number of ORFs and frequency of OCL genes present, were investigated. OCL sequences were found to range between 6 and 10 kb (mean 8 kb) in total length (Fig. 4a, Table 1) and include between 6 and 11 (mean 9) ORFs per locus (Fig. 4b, Table 1). A gene presence/absence matrix categorizing all ORFs across 22 OCLs into ‘homology groups’ based on an 85 % aa identity cut-off was generated using Roary to calculate how many ORFs were shared amongst OCL types. A total of 57 genes (homology groups) were detected across the 22 OCLs and, of these, 40.3 % were found to be unique to a single locus type (Fig. 4c). Only 1.8 % of genes were found to be present in more than 75 % of all OCL types, with *gtrOC1* being the only gene present in all loci.

As the annotations of most genes included in the OCL reference database were originally assigned based on protein family (Pfam) and clan assignments from searches conducted in 2014 [14], representative sequences for each homology group were subjected to hmmer to re-assess these assignments (Table 2). Of the 57 OCL genes, 37 were found to predict glycosyltransferases (GtrOC). The majority of these belong to glycosyltransferase-associated Pfams, confirming their designation as GtrOC proteins. However, consistent with previous searches, five proteins (GtrOC1, GtrOC14, GtrOC19a, GtrOC19b and GtrOC22) were found to belong to the Mito_fiss_Elm1 (PF06258.14) mitochondrial fission protein family that has no known function in bacteria [14]. One GtrOC protein (GtrOC7), not previously assigned to any family, was found to include domains for Stealth_CR1–CR4 families associated with CPS phosphotransferases. Two further proteins (GtrOC6 and GtrOC28) could not be assigned to a defined protein family, though GtrOC28 was previously found to belong to the Glyco_tranf_2_5 glycosyltransferase family in the previous study [14].

**Table 2.** Predicted function of proteins encoded by OCL genes

Protein	Predicted function	Protein family (Pfam)*	Clan*
GtrOC1	Hypothetical†	Mito_fiss_Elm1 (PF06258.14)	CL0113
GtrOC2	Glycosyltransferase	Glyco_trans_1_2 (PF13524.9)	CL0113
GtrOC3	Glycosyltransferase	Glycos_transf_2 (PF00535.29); Glycos_transf_2_4 (PF13704.9)	CL0110
GtrOC4	Glycosyltransferase	Glycos_transf_1 (PF00534.23); Glyco_trans_1_4 (PF13692.9)	CL0113
GtrOC5	Glycosyltransferase	Glyco_transf_25 (PF01755.20)	CL0110
GtrOC6	Hypothetical†,‡	–	–
GtrOC7	Hypothetical†,‡	Stealth_CR1 (PF17101.8); Stealth_CR2 (PF11380.11)	–
GtrOC8	Glycosyltransferase	Glyco_transf_25 (PF01755.20)	CL0110
GtrOC9	Glycosyltransferase	Gly_transf_sug (PF04488.18)	CL0110
GtrOC10	Glycosyltransferase	Gly_transf_sug (PF04488.18)	CL0110
GtrOC11	Glycosyltransferase	Glycos_transf_1 (PF00534.23); Glyco_trans_1_4 (PF13692.9)	CL0113
GtrOC12	Glycosyltransferase	Glycos_transf_1 (PF00534.23); Glyco_trans_1_4 (PF13692.9)	CL0113
GtrOC13	Glycosyltransferase	Glycos_transf_1 (PF00534.23); Glyco_trans_1_4 (PF13692.9)	CL0113
GtrOC14	Hypothetical†	Mito_fiss_Elm1 (PF06258.14)	CL0113
GtrOC15	Glycosyltransferase	Glycos_transf_1 (PF00534.23); Glyco_trans_1_4 (PF13692.9)	CL0113
GtrOC16	Glycosyltransferase	Glyco_transf_8 (PF01501.23); Glyco_transf_8C (PF08437.13)	CL0110
GtrOC17	Glycosyltransferase	Glyco_trans_1_2 (PF13524.9)	CL0113
GtrOC18	Glycosyltransferase	Glycos_transf_1 (PF00534.23); Glyco_trans_1_4 (PF13692.9)	CL0113
GtrOC19a	Hypothetical†	Mito_fiss_Elm1 (PF06258.14)	CL0113
GtrOC19b	Hypothetical†	Mito_fiss_Elm1 (PF06258.14)	CL0113
GtrOC20	Glycosyltransferase	Glycos_transf_2 (PF00535.29); Glyco_tranf_2_3 (PF13641.9)	CL0110
GtrOC21	Glycosyltransferase	Glyco_trans_1_2 (PF13524.9); DUF3880 (PF12996.10)	CL0113
GtrOC22	Hypothetical†	Mito_fiss_Elm1 (PF06258.14)	CL0113
GtrOC23	Glycosyltransferase	Glycos_transf_1 (PF00534.23); Glyco_trans_1_4 (PF13692.9)	CL0113
GtrOC24	Glycosyltransferase	Glycos_transf_2 (PF00535.29); Glyco_tranf_2_3 (PF13641.9)	CL0110
GtrOC25	Glycosyltransferase	Glycos_transf_1 (PF00534.23); Glyco_trans_1_4 (PF13692.9)	CL0113
GtrOC26	Glycosyltransferase	Glycos_transf_2 (PF00535.29); Glyco_tranf_2_3 (PF13641.9)	CL0110
GtrOC27	Glycosyltransferase	Gly_transf_sug (PF04488.18); Caps_synth (PF05704.15)	CL0110
GtrOC28	Hypothetical†, §	–	–
GtrOC29	Glycosyltransferase	Glyco_transf_25 (PF01755.20)	CL0110
GtrOC30	Glycosyltransferase	Glyco_transf_25 (PF01755.20)	CL0110
GtrOC31	Glycosyltransferase	Glyco_transf_25 (PF01755.20)	CL0110
GtrOC32	Glycosyltransferase	Glycos_transf_2 (PF00535.29); Glyco_tranf_2_3 (PF13641.9)	CL0110
GtrOC33	Glycosyltransferase	Gly_transf_sug (PF04488.18); Caps_synth (PF05704.15)	CL0110
GtrOC34	Glycosyltransferase	Glycos_transf_2 (PF00535.29); Glyco_tranf_2_3 (PF13641.9)	CL0110
GtrOC35	Glycosyltransferase	Glycos_transf_1 (PF00534.23); Glyco_trans_1_4 (PF13692.9)	CL0113
GtrOC36	Glycosyltransferase	Glyco_transf_25 (PF01755.20)	CL0110
Ahy	Acetylhydrolase	Lipase_GDSL_2 (PF13472.9)	CL0264
AtrOC1	Acyltransferase	Acyl_transf_3 (PF01757.25)	CL0316
Glf	UDP-galactopyranose mutase	GLF (PF03275.16); NAD_binding_8 (PF13450.9)	CL0063
HtrL	Hypothetical	HtrL_YibB (PF09612.13)	–
Orf1 (Ghy)	Hypothetical/glycosylhydrolase	–	–
Orf2	Hypothetical	–	–
Orf3	Hypothetical	DUF707 (PF05212.15)	–
Orf4	Hypothetical	Capsule_synth (PF05159.17)	CL0113
Pda1	Polysaccharide deacetylase	Polysacc_deac_1 (PF01522.24)	CL0158
Pda2	Polysaccharide deacetylase	Polysacc_deac_1 (PF01522.24)	CL0158
PtrOC1	Pyruvyltransferase	PS_pyruv_trans (PF04230.16)	CL0113
QdtB	dTDP-4-oxo-6-deoxy-d-glucose 3,4-oxoisomerase/acetyltransferase	DegT_DnrJ_EryC1 (PF01041.20); Aminotran_1_2 (PF00155.24)	CL0061
QdtE	Aminotransferase	FdtA (PF05523.14); Hexapep (PF00132.27)	CL0029
RmlA	Glucose-1-phosphate thymidylyltransferase	NTP_transferase (PF00483.26); NTP_transf_3 (PF12804.10)	CL0110
RmlB	dTDP-glucose 4,6-dehydratase	GDP_Man_Dehyd (PF16363.8); Epimerase (PF01370.24)	CL0063
RmlC	dTDP-4-dehydrorhamnose 3,5-epimerase	dTDP_sugar_isom (PF00908.20)	CL0029
RmlD	dTDP-6-deoxy-l-lyxo-4-hexulose reductase	RmlD_sub_bind (PF04321.20);Epimerase (PF01370.24)	CL0063
WecB	UDP-*N*-acetylglucosamine 2-epimerase	Epimerase_2 (PF02350.22)	CL0113

*Search conducted November 2022 using hmmer with the Pfam database. Only two predicted families of the highest confidence matches are reported per protein. –, Not known.

†Probable glycosyltransferases, as previously predicted [14]. GtrOC annotations retained.

‡GtrOC6 and GtrOC7 previously reported as glycosyltransferases as seven Gtrs are predicted to be required for the synthesis of OC1 [13].

§Searches conducted in May 2014 [14] predicted that GtrOC28 belongs to Pfam Glyco_transf_2_5 (CL0110). Updated analysis has revealed no detectable Pfam motifs.

In addition to the 37 genes encoding GtrOC proteins, 10 genes were found to encode enzymes predicted to be responsible for nucleotide-linked sugar biosynthesis (either *rmlBDAC* for dTDP-l-rhamose, *rmlBA/qdtBE* for 3-acetamido-3,6-dideoxy-d-galactose, *wecB* for *N*-acetyl-d-mannosamine and *glf* for d-galactofuranose). Two further genes are for predicted polysaccharide deacetylases (Pda1 and Pda2), one for a predicted acylhydrolase (Ahy), one for a putative acetyltransferase (AtrOC1), one for a putative pyruvyltransferase (PtrOC1) and five predicting proteins of unknown function (ORF1–4 and HtrL) (Table 2, Fig. 4d). The *orf1* gene, found in all OCLs in group A, was originally predicted to encode a glycosylhydrolase and annotated as *ghy* in the earliest study on the OCL [11]. However, this was since reassigned as *orf1* based on updated Pfam searches [14].

## Discussion

In this study, we report an update to the *A. baumannii* OCL reference sequence database to include four OCL types (OCL13–OCL16) characterized since the release of the original database [18, 19] and six new OCLs (OCL17–OCL22) identified amongst available sequenced genomes, providing a total of 22 distinct OCL types. Analysis of the distribution of OCL types demonstrated that OCL1 was present in a large proportion (73.6%) of available sequenced genomes. However, the over-representation of OCL1 may be skewed by bias in the sampling of isolates for sequencing studies, particularly those belonging to GC2. Though this analysis also showed that most common OCL types had been accounted for in the original version of the database [12], the updated version encompasses a further ten OCLs, increasing its utility for detecting a broader variety of OCL types in *A. baumannii *.

While new OCLs were uncovered in this study, we cannot exclude the possibility that further novel types may be present in the 2834 genomes that were not investigated due to the locus being detected across >1 contig. The finding of the OCL in two or more contigs can be due to the occurrence of ISs interrupting the locus [12, 13], and several previous studies have demonstrated IS interruption of OCL, particularly in the over-represented OCL1 locus [9, 11, 12, 15]. The presence of a composite transposon interrupting OCL1 has also been described previously [9], and here we report further interruptions of OCL1 by two novel transposon sequences. It is not clear what properties are conferred by these transposons or the effect that these might have on the LOS. However, as the interruption of specific genes in OCL1 has been shown to lead to significant truncation of the LOS structure [15], the characterization of the specific site of insertion(s) of either ISs or transposons at the OCL is recommended when assessing LOS structure or virulence properties associated with LOS in *A. baumannii *.

All novel types identified in this study resembled known OCLs and could be classified into group A or group B, the two major OCL configurations (Fig. 1) previously defined by the presence of common genes adjacent to *ilvE* [14]. Though no new variations to the general arrangement of the OCL were uncovered, two subgroups within group B could be observed, distinguished by the presence of either *gtrOC13* or *gtrOC18* immediately adjacent to *pda2*. Within group A, the newly identified OCL18 locus was the only type found to vary in the genetic content of region 1. OCL18 does not include *pda1* or *gtrOC3* but was assigned to group A as it carries *gtrOC1*, *gtrOC2* and *orf1* like other locus types in this group. Apart from OCL18, which may represent a subgroup within group A, all other variation between OCL types was observed in region 2 (Fig. 1).

The genetic repertoire of the OCL was found to be significantly less diverse than what has been observed at the *A. baumannii* KL, which includes a repertoire of 681 genes across 237 KL types, for which 272 genes are predicted to encode glycosyltransferases [13]. However, like the KL, the OCL is considered a recombination ‘hotspot’ [8, 30], and most OCL genes are either unique to a single OCL or present in less than a quarter of all OCL types. Re-assessment of the annotations previously assigned to all genes based on the detection of protein domains/motifs and predicted functional roles [11, 14] revealed that some GtrOC proteins do not belong to a known glycosyltransferase family. Previous blastp searches of these sequences had detected protein homologues variously annotated as glycosyltransferases or hypothetical [11, 14]. Furthermore, it had been suggested that the presence of seven putative GtrOC proteins encoded by OCL1 (three of which do not belong to a known Pfam) corresponds with the requirement for seven glycosyltransferases to synthesize the known OC1 structure [11, 14]. Hence, the GtrOC annotations for these proteins have been retained in the database, but may change once experimental data confirming their functions becomes available.

Across *Acinetobacter* spp., variation in gene content can also be observed just outside the OCL, where a putative *waaL* O-antigen ligase gene can be found in some genomes, located between *aspS* and an unknown ORF that is immediately upstream of a gene encoding a TonB-dependent receptor [14]. However, in this study, we did not detect any genes predicted to encode a WaaL ligase inserted at this location in the studied *A. baumannii* genomes. The absence of a *waaL* gene in the *A. baumannii* genome was first demonstrated in 2013 [11], and later confirmed using a wider variety of *A. baumannii* genome sequences in the following year [14]. While two *waaL*-like genes were detected in some *A. baumannii* genomes [6, 30], these were later shown to encode ligases that link CPS oligosaccharide units to either the type IV pilus or proteins through O-linked glycosylation [31, 32]. Absence of an O-antigen was also experimentally confirmed for isolate ATCC 17978 by the lack of an O-antigen banding pattern on Western blots using an anti-lipid A antibody [15]. Hence, all available evidence to date provides strong support that *A. baumannii* produces LOS, with structural variation predominately seen in the OC.

Variation at the OCL in clonal lineages has been reported extensively in previous studies [e.g. 8, 9, 11, 12, 14, 30, 33–35]. In our previous large-scale analysis of >3600 genome sequences available at that time, we detected six OCL types in ST1, and four OCL types in both ST2 and ST25 [12]. Here, we report the finding of an additional four OCLs in ST2, bringing the total to eight, with OCL1 being the most common. No additional OCL types were found in the ST1 lineage, though OCL1 also remains the most predominant type in available sequenced genomes belonging to this clone. ST25 was found to include three additional OCLs, but surprisingly does not carry OCL1. For the other over-represented STs, only one OCL type was detected in ST3, ST10, ST32 and ST78, two OCLs in ST499, and four OCLs in ST79. While we have detected additional variation at the OCL in clonal isolates, it is likely that further OCL forms will be observed amongst isolates belonging to the same ST as more genomes are sequenced and released. This highlights the importance of including OCL typing in epidemiological studies on *A. baumannii* clonal lineages.

## Supplementary Data

Supplementary material 1

Supplementary material 2
